# Bosutinib‐Associated Cardiac Tamponade: Late‐Onset Treatment‐Emergent Adverse Event—A Case Report

**DOI:** 10.1155/cric/6696107

**Published:** 2026-05-03

**Authors:** Mehmet Ası Oktan, Hüseyin Dursun, Yelda Deligöz Bildacı, Berfu Korucu, Cihan Heybeli, Caner Çavdar, Serpil Müge Değer

**Affiliations:** ^1^ Faculty of Medicine, Department of Internal Medicine, Division of Nephrology, Dokuz Eylul University, Izmir, Turkey, deu.edu.tr; ^2^ Department of Cardiology, Dokuz Eylül University School of Medicine, Izmir, Turkey, deu.edu.tr

**Keywords:** acute kidney injury, bosutinib, cardiac tamponade, chronic myeloid leukemia, tyrosine kinase inhibitors

## Abstract

Fluid retention due to tyrosine kinase inhibitor administration might lead to pericardial effusion and right heart failure. Here, we present the case of a 63‐year‐old man with subacute cardiac tamponade complicated with heart failure, acute kidney injury, and generalized edema due to the bosutinib treatment.

## 1. Background

Tyrosine kinase inhibitors (TKIs) have become a cornerstone in treating many malignancies in the oncology field [1]. Chronic myeloid leukemia (CML) is a myeloproliferative disorder that occurs after chromosomal translocation and carries the tyrosine kinase activity of the BCR::ABL1 oncoprotein. Bosutinib is a second‐generation TKI that targets the BCR::ABL1 fusion gene product and is indicated in chronic phase CML resistant or intolerant to imatinib treatment [2].

Bosutinib‐associated side effects are mainly reported as gastrointestinal symptoms such as diarrhea, nausea, vomiting, and liver test abnormalities that can be generally controlled by dose interruptions or reductions [2–3]. Bosutinib has a more selective molecular target spectrum of action than other TKIs. Since it has less effect on platelet‐derived growth factor and c‐KIT, cardiovascular complications are less encountered in clinical practice compared with other TKIs [4]. Heart failure and ischemic events due to bosutinib treatment have previously been reported in the literature and mainly patients with pre‐existing cardiac risk factors. Severe fluid retention requiring permanent discontinuation of the drug is rare (< 1%), and the cases were manifested as pericardial and pleural effusions, as well as pulmonary and/or peripheral edema [5]. In the randomized clinical trial (BFORE Trial) including 268 adult patients with newly diagnosed CML, it has been reported that in the bosutinib treatment group, three patients (1.1%) experienced severe fluid retention, one patient severe pericardial effusion, and two patients severe pleural effusion [6]. Whereas in newly diagnosed or treatment‐naive patients Grade 3 or 4 fluid retention incidence is around 1%, on the other hand as stated in recent cardio‐oncology guideline patients with CML who were resistant or intolerant to prior therapy, Grade 3 or 4 fluid retention was reported as much as 10% [3, 7].

Here, we present the case of a patient with chronic‐phase CML who was resistant and intolerant to prior TKIs; following the switch to bosutinib treatment, the clinical course was complicated by late‐onset subacute cardiac tamponade, recurrent pericardial effusion, and massive pulmonary and peripheral edema.

## 2. Case Presentation

**Figure 1:**
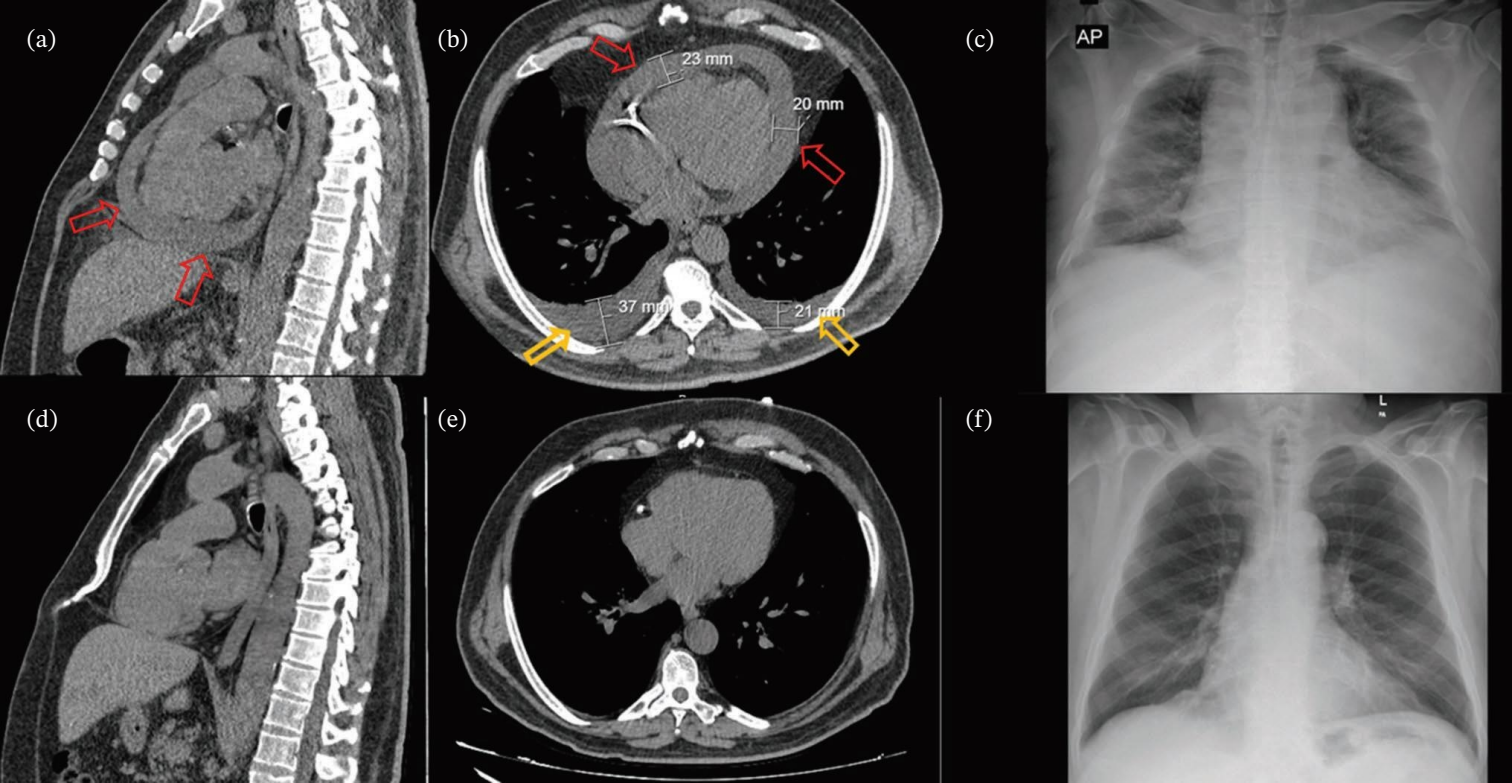
Computed thorax tomography imaging: Hospital admission (a–b), 1 year before (d–e). (a) Pericardial fluid accumulation indicated with red arrows in the sagittal section. (b) Pericardial fluid (red arrows) and pleural effusion (yellow arrow) shown in the transverse section. (c) Posteroanterior chest radiography with cardiomegaly and interstitial edema at hospital admission. (d–e) No pericardial or pleural effusion in previous computed thorax tomography. (f) Normal chest radiography at hospital discharge.

A 63‐year‐old man was diagnosed with CML 7 years ago. He had a comorbidity of diabetes mellitus and coronary artery disease. He was on medications including perindopril, ticagrelor, metoprolol, atorvastatin, and basal‐bolus insulin therapy due to coronary artery disease and diabetes mellitus. After the diagnosis of CML, he was initially treated with imatinib for 4 years and switched to nilotinib due to the progression of the primary disease. Subsequently, another switch to bosutinib (500 mg/day) was made due to metabolic complications after 2 years. At the 10th month of bosutinib therapy, he was admitted to the hospital complaining of sudden onset of dyspnea, bilateral leg edema, and weight gain in the last several days. Physical examination revealed tachycardia (116 beats per minute), hypotension (75/44 mmHg), hypoxemia (Sp0_2_, 84), hepatomegaly, and severe bilateral pitting leg edema. Laboratory data showed an increase in serum creatinine of 2.9 mg/dL (baseline, 1.5 mg/dL), sodium of 131 mEq/L, serum potassium of 5.5 mEq/L, and bicarbonate 20 mEq/L. Serum albumin, thyroid function tests, troponin levels, B‐type natriuretic peptide (BNP) levels, and urine protein excretion were within the normal range. Computed thorax tomography showed new, severe bilateral lung edema and pericardial effusion compared with the previous scans (Figure 1). Transthoracic echocardiography documented severe cardiac tamponade. Collapse of the right‐sided cardiac chambers was observed. A total of 1 L of serosanguinous fluid was drained over 24 h by parasternal pericardiocentesis. Norepinephrine was started, and ultrafiltration was also performed for generalized volume overload. Vital signs became stable right after pericardiocentesis, and spontaneous diuresis started. Bosutinib treatment was stopped. One week after his hospitalization, his urine output decreased again. Repetitive echocardiography demonstrated the recurrent pericardial effusion. Surgically draining by pericardial window technique and pericardial biopsy were performed. Pericardial fluid samples were analyzed for biochemical analysis, gram stain, acid‐fast bacillus stain‐culture, pH, adenosine deaminase level, and cytologic examination (Table 1). Testing for autoimmune serology yielded negative results. After the minimally invasive surgery, hemodynamic stabilization was achieved, and urine output increased spontaneously. Ultrafiltration or dialysis was no longer required. Treatment with methylprednisolone 0.5 mg/kg/day and colchicine 0.5 mg/day was started due to the refractory pericardial effusion and acute pericarditis [8, 9]. He was discharged from the hospital with his dry weight.

**Table 1:** Laboratory and pathologic examination of pericardial fluid and biopsy.

pH	7.35
Glucose level	171 mg/dL (pericardial fluid to serum ratio > 0.5)
Triglycerides	95 mg/dL
White cell count	600/*μ*L, polymorphonuclear predominant (61%)
Total protein	3.58 g/dL (pericardial fluid to serum ratio > 0.5)
Albumin	1.95 g/dL (pericardial fluid to serum ratio > 0.5)
Lactate dehydrogenase	188 U/L (pericardial fluid to serum ratio > 0.6)
Microbiologic gram stain and cultures	Negative
Acid fast smear and tuberculous culture	Negative
Adenosine deaminase	10.2 U/L
Cytology	Nonneoplastic‐mixed inflammatory cells
Biopsy	Acute pericarditis characterized by fibrinopurulent exudate and occasional granulation tissue formation and fibrous thickening. Immunohistochemical examinations revealed that macrophages positively stained with CD163 were abundant. Any findings suggestive for epithelial malignancy were not found.

## 3. Outcome and Follow‐Up

**Figure 2:**
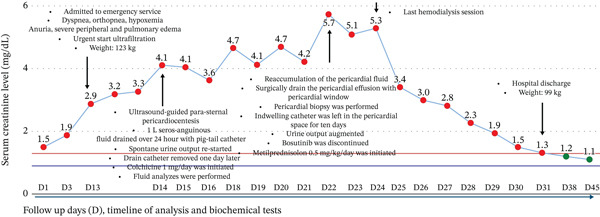
The clinical course of the patient.

The glucocorticoid was tapered and discontinued within 6 weeks. In line with expert panel recommendations, it was decided to restart bosutinib at a lower dose (300 mg/day), after treatment was interrupted for 4 weeks [10]. No evidence of increased CML activity was observed during this period. No pericardial effusion was detected in the control echocardiographic imaging, and the patient was observed in a stable molecular response (BCR::ABL1 level is < 0.1%) with normal renal functions (Figure 2). Initially, CML activity and cardiac function were monitored monthly; subsequently, the intervals between follow‐up assessments were extended.

## 4. Discussion

Here, we have presented a case of cardiac tamponade along with generalized severe edema following bosutinib treatment. It has been reported that bosutinib, which has a narrow protein kinase effect spectrum, causes cardiovascular side effects less frequently compared with the other TKIs [11]. Since there is a need for urgent intervention in our case, the degree of cardiac adverse event associated with bosutinib is defined as “Grade 4” according to the American National Cancer Instıtute Common Terminology Criteria for Adverse Events [12]. Even though our patient was using the bosutinib treatment for 10 months, a fatal treatment‐emergent adverse event was deemed worthy of reporting.

Bosutinib treatment is reported to carry a low risk of cardiovascular side effect profile compared with imatinib according to the Phase 3 trials (BELA and BFORE trial). It should be noted that the observation period of these studies was limited to 12 months, which is sufficient to demonstrate drug efficacy; however, longer observation is needed to describe adverse events of any molecule [5, 6]. In clinical practice, clinicians should keep in mind fatal treatment‐emergent adverse events by monitoring the patients under TKI treatment.

The Naranjo Scale is used to predict adverse reactions due to the drug administration rather than other factors (Table S1) [13].

In this situation, the fact that there was no fluid around the heart (pericardial effusion) or lungs (pleural effusion) seen on imaging tests 1 year before starting bosutinib treatment is considered a positive sign regarding the risk of severe fluid retention and cardiac tamponade (3 points), and no alternative causes (such as malign infiltration or infection) were detected from recent fluid analysis (2 points). In addition, there are previous reports on this adverse reaction (1 point) [14]. According to the Naranjo algorithm, our patient is categorized in the “Probable” section with a total of 6 points, suggesting the presence of bosutinib‐associated serious adverse events.

The mechanistic relationship between TKI‐induced cardiovascular toxicity is not fully understood. TKIs affect not only cancer cells but also healthy ones, which are conclusive for side‐effect mechanisms [15]. Nonselective inhibition of protein kinase is involved as “off‐target inhibition” [16]. Bosutinib, the new generation of TKI with a narrowed molecular target, seems to have a lower off‐target effect compared with the previous ones. However, unexpected severe side effects might occur in the presence of pre‐existing cardiovascular diseases. Hence, clinicians should closely monitor patients with diabetes mellitus, obesity, and a history of smoking.

The common strategy for managing adverse events due to TKIs is dose interruption or reduction. Patients who develop signs and symptoms of pleural/pericardial effusions or pulmonary edema during bosutinib treatment should undergo a detailed evaluation to exclude other disorders. Therapeutic drainage and glucocorticoids should be considered in urgent treatment. Bosutinib might be prescribed at a lower dose (200–300 mg/day) after resolution of the volume overload [10]. Subsequently, the dropping down dose should be escalated by BCR::ABL1 mutation analysis and patient tolerance profile as recommended by European experts′ guidelines [2]. Moreover, one should be alert for drug interactions with TKIs and be careful in combined use of drugs that affect hepatic metabolism through CYP activity.

In conclusion, we reported a case of bosutinib‐associated cardiac tamponade along with generalized edema that required urgent drainage and resolved after discontinuation of the drug. The underlying mechanism for effusion side effects appears to be due to the off‐target effects of different TKIs. The assessment of cardiovascular risk is essential before starting TKI therapy. Prospective studies to evaluate the safety of TKIs in patients with cardiac disease are needed.

## 5. Learning Points

TKIs are drugs that change the paradigm in cancer treatment, but although rare, they can cause fatal side effects such as massive fluid retention. Clinicians should be aware that side effects may develop even months later.

## Author Contributions

Concept: M.A.O. and H.D.; design: M.A.O. and C.H.; supervision: S.M.D. and C.Ç.; resources: M.A.O., C.H., and Y.D.B.; materials: M.A.O., B.K., and Y.D.B.; data collection and/or processing: M.A.O., B.K., and H.D.; analysis and/or interpretation: M.A.O., C.H., C.Ç., and S.M.D.; literature search: M.A.O.; writing manuscript: M.A.O. and S.M.D.; critical review: C.H., C.Ç., and S.M.D.

## Funding

No funding was received for this manuscript.

## Ethics Statement

Ethical approval was received from the local ethics committee.

## Consent

Informed consent was obtained from all individual participants included in the case report. Approval for publication was obtained from all authors.

## Conflicts of Interest

The authors declare no conflicts of interest.

## Supporting information


**Supporting Information** Additional supporting information can be found online in the Supporting Information section. Table S1: The Naranjo Adverse Drug Reaction Probability Scale used to assess drug toxicity.

## Data Availability

Clinical information and hospital records for this case report will be shared upon a request from the corresponding author.
