# Supplementary material for: Bosutinib‐Associated Cardiac Tamponade: Late‐Onset Treatment‐Emergent Adverse Event—A Case Report
Source: Case Rep Cardiol. 2026 May 3;2026:6696107. doi: 10.1155/cric/6696107 (PMC13136675; doi:10.1155/cric/6696107)
Supplement: Supplementary file 1 — Supporting Information Additional supporting information can be found online in the Supporting Information section. Table S1: The Naranjo Adverse Drug Reaction Probability Scale used to assess drug toxicity. [file CRIC-2026-6696107-s001.docx]

### Supplemental Table 1. Naranjo Algorithm- Adverse Drug Reaction Probability Scale

| **Question** | **Yes** | **No** | **Do not Know** | **Score** |
| --- | --- | --- | --- | --- |
| 1. Are there previous conclusive reports on this reaction? | **+1** | **0** | **0** |  |
| 1. Did the adverse event appear after the suspected drug was administered? | **+2** | **-1** | **0** |  |
| 1. Did the adverse event improve when the drug was discontinued or a specific antagonist was administered? | **+1** | **0** | **0** |  |
| 1. Did the adverse event reappear when the drug was readministered? | **+2** | **-1** | **0** |  |
| 1. Are there alternative causes that could on their own have caused the reaction? | **-1** | **+2** | **0** |  |
| 1. Did the reaction reappear when a placebo was given? | **-1** | **+1** | **0** |  |
| 1. Was the drug detected in blood or other fluids in concentrations known to be toxic? | **+1** | **0** | **0** |  |
| 1. Was the reaction more severe when the dose was increased or less severe when the dose was decreased? | **+1** | **0** | **0** |  |
| 1. Did the patient have a similar reaction to the same or similar drugs in any previous exposure? | **+1** | **0** | **0** |  |
| 1. Was the adverse event confirmed by any objective evidence? | **+1** | **0** | **0** |  |
| \| **Total Score** \| **Interpretation of Scores** \| \| --- \| --- \| \| **≥9** \| **Definite**. The reaction (1) followed a reasonable temporal sequence after a drug or in which a toxic drug level had been established in body fluids or tissues, (2) followed a recognized response to the suspected drug, and (3) was confirmed by improvement on withdrawing the drug and reappeared on reexposure. \| \| **5-8** \| **Probable**. The reaction (1) followed a reasonable temporal sequence after a drug, (2) followed a recognized response to the suspected drug, (3) was confirmed by withdrawal but not by exposure to the drug, and (4) could not be reasonably explained by the known characteristics of the patient’s clinical state. \| \| **1-4** \| **Possible**. The reaction (1) followed a temporal sequence after a drug, (2) possibly followed a recognized pattern to the suspected drug, and (3) could be explained by characteristics of the patient’s disease. \| \| **≤0** \| **Doubtful**. The reaction was likely related to factors other than a drug. \| | **Total Score:** | | |  |
